# Adsorption of Indigo Carmine onto Zn–Cu–Fe layered triple hydroxides and oxides: a comparative investigation of removal mechanisms and matrix effects

**DOI:** 10.1039/d6ra03446b

**Published:** 2026-06-03

**Authors:** Mohamed Farag, Sami A. Al-Hussain, Arafat Toghan, Emad M. Masoud, Ashraf A. Mohamed, Mohamed A. Ahmed, Hoda A. Ahmed, Mahmoud A. Ahmed

**Affiliations:** a Chemistry department, Faculty of Science, Ain Shams University Cairo-11566 Egypt mahmoudmahmoud_p@sci.asu.edu.eg; b Chemistry Department, College of Science, Imam Mohammad Ibn Saud Islamic University (IMSIU) Riyadh 11623 Saudi Arabia; c The Higher Institute for Optics Technology, Culture and Science CITY Cairo 17361 Egypt; d Department of Chemistry, Faculty of Science, Islamic University of Madinah Madinah 42351 Saudi Arabia emad.youssef@iu.edu.sa; e Department of Chemistry, College of Science in Yanbu, Taibah University Yanbu Governorate Saudi Arabia

## Abstract

Layered triple hydroxides and their calcined oxides offer distinct adsorption pathways for anionic pollutants, yet how thermal transformation redirects these mechanisms, particularly for bulky dye molecules, remains poorly understood. This study addresses this gap by synthesizing a Zn–Cu–Fe layered triple hydroxide (LTH) *via* coprecipitation and its calcined oxide (LTO) at 500 °C, comparing their behavior toward Indigo Carmine (IC) through integrated characterization, batch adsorption studies, and mechanistic analysis. Both materials were comprehensively examined using FTIR, FESEM-EDS, HRTEM, BET, and zeta potential, revealing that LTH possesses a high surface area with a well-ordered layered structure, while LTO comprises intimately mixed ZnO, CuO, and ZnFe_2_O_4_ phases with reduced surface area but enlarged mesopores and magnetic properties. Adsorption studies demonstrate that LTH achieves superior removal through monolayer coverage on homogeneous external surfaces following pseudo-first-order kinetics, while LTO exhibits lower capacity with faster initial kinetics and heterogeneous binding across its multiphase surface. Thermodynamic analysis confirms endothermic physisorption for both. Crucially, mechanistic elucidation through post-adsorption FTIR and FESEM reveals that despite LTH's layered architecture, IC molecules are too large to access the interlayer galleries; uptake instead proceeds *via* electrostatic attraction to external surfaces and edge-localized carbonate displacement. For LTO, a synergistic mechanism operates, pore-filling within the macroporous network, surface complexation on coordinatively unsaturated metal centers, and memory effect reconstruction that partially reforms layered domains incorporating additional dye. Maximum Langmuir capacities of LTH and LTO for IC at 35 °C, were 71.34 and 69.15 mg g^−1^. The PFO kinetic model best described IC adsorption on both materials with q_exp_ of 48.6 mg g^−1^. The BET surface areas of LTH and LTO were 150.14 and 21.70 m^2^ g^−1^, with total pore volumes of 0.2645 and 0.1982 cm^3^ g^−1^ at *p*/*p*^0^ = 0.990 and the mean pore diameters were 7.05 and 36.53 nm, for LTH and LTO, respectively. These values provide a clearer snapshot of the performance and mechanistic differences between LTH and LTO. Both materials maintain IC selectivity in binary dye systems with minimal interference from NaCl or humic acid. The contrasting pathways demonstrate that calcination fundamentally transforms the adsorption mechanism, offering complementary strategies for anionic dye remediation.

## Abstract 2

Layered triple hydroxides and their calcined oxides offer distinct adsorption pathways for anionic pollutants, yet how thermal transformation redirects these mechanisms, particularly for bulky dye molecules, remains poorly understood.

## Graphical abstract

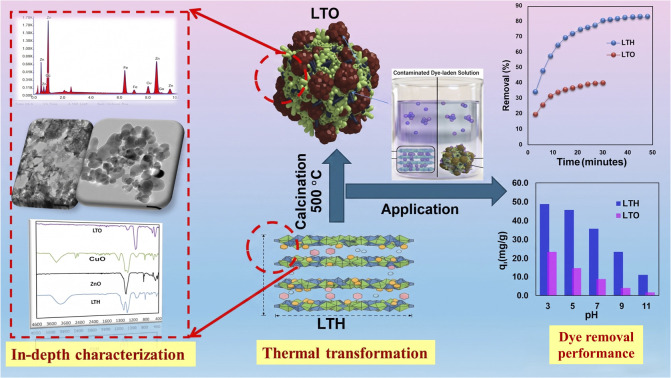

## 1 Introduction

Rapid industrialization, urbanization, and population growth have placed mounting pressure on global water resources through continuous discharge of diverse pollutants—including heavy metals, pesticides, pharmaceuticals, and synthetic dyes—into aquatic ecosystems.^1–3^ Among these, synthetic dyes are particularly concerning due to their extensive industrial use. With global annual production exceeding 700 000 tons, approximately 10–15% of synthetic dyes enter water bodies during manufacturing and textile processing.^4,5^ Their complex aromatic structures confer remarkable stability, making them resistant to natural degradation by light, heat, or microbial action.^6^ This persistence reduces light penetration in water, inhibiting photosynthesis in aquatic plants and algae, thereby disrupting the entire food chain 7. Additionally, many dyes and their metabolites exhibit toxic, carcinogenic, and mutagenic effects on aquatic organisms and humans.^8,9^ Various treatment methods including physical, chemical, and biological approaches have been developed, but face limitations such as high costs, energy intensity, secondary waste, or incomplete removal.^10^

Adsorption has emerged as a preferred alternative due to its simplicity, cost-effectiveness, high efficiency, and ability to remove pollutants without generating harmful by-products.^11,12^ A robust adsorbent is characterized by several key features including high specific surface area, appropriate porosity, abundant active sites, surface functionality, chemical stability, and regeneration capacity.^13,14^ The nature of interactions between adsorbent and adsorbate determines the adsorption mechanism, which broadly falls into two categories. Physisorption involves weak van der Waals forces, electrostatic interactions, or hydrogen bonding, characterized by low enthalpy changes, reversibility, multilayer formation, and rapid equilibrium attainment.^15–17^ In contrast, chemisorption entails stronger chemical bond formation through electron transfer or sharing, resulting in higher enthalpy changes, irreversibility, monolayer coverage, and slower kinetics.^17,18^ The selection of adsorbent material governs which mechanism predominates. A wide spectrum of adsorbents has been developed for dye removal, ranging from conventional carbon-based materials such as activated carbon, biochar, carbon nanotubes, and graphene oxide—which offer high surface areas and versatile surface chemistry—to emerging materials including metal–organic frameworks (MOFs) with exceptional porosity, and MXenes, two-dimensional transition metal carbides exhibiting hydrophilic surfaces and metallic conductivity.^19–22^ Other materials including zeolites, clay minerals, silica, and metal oxides have also been extensively investigated.

Layered triple hydroxides (LTHs) belong to the family of hydrotalcite-like compounds, which trace their origins to the early 1840s in Sweden, though their definitive formula was not established until 1915 when Manasse described natural mineral hydrotalcite as [Mg_6_Al_2_(OH)_16_]CO_3_·4H_2_O.^23–25^ These materials possess a unique layered structure derived from brucite (Mg(OH)_2_), where magnesium ions are octahedrally coordinated by hydroxyl groups, forming neutral stacked layers. In LTHs, partial isomorphous substitution of divalent cations (M^2+^) by trivalent cations (M^3+^) generates a net positive charge on the hydroxide layers, as each trivalent cation contributes an additional positive charge.^25,26^ To maintain electroneutrality, charge-compensating anions along with water molecules occupy the interlayer gallery, resulting in the general formula [M(II)_1−*x*_{M(III)_1−*γ*_M(III)_*γ*_}_*x*_(OH)_2_]*A*_*x*/*n*_^*n*–^·*m*H_2_O, where M(II) and M(III) represent divalent and trivalent metals, *A*^*n*−^ denotes the *n*-valent interlayer anion, and *x* and *y* indicate the proportion of trivalent metal cations.^25–27^ These positively charged layers are separated by interlayer regions containing water molecules and anions such as carbonates or nitrates, which balance the charge and can be exchanged to modify material properties.^28,29^ The layers are held together by hydrogen bonds, preserving the porous, high-surface-area characteristics essential for contaminant interaction in wastewater treatment.^30^ The morphology of LTHs typically appears as two-dimensional hexagonal nanoflakes with crystalline structure, as revealed by TEM analysis showing average particle sizes ranging from 63.50 to 139.09 nm depending on metal ratios, with lattice spacings around 0.151 nm confirming their 2D nanoparticulate nature.^31^ HRTEM analysis of LTH-based composites has revealed 2D/2D plate-like structures with interlayer spacings of 0.26, 0.18, and 0.16 nm corresponding to specific peak positions, while SAED patterns confirm their polycrystalline nature.^30^ The flexible composition of LTHs allows incorporation of various metal combinations, such as Zn, Cu, and Fe in the present ternary system, enabling tuning of layer charge density, surface chemistry, and affinity toward specific pollutants. Unlike conventional layered double hydroxides (LDHs) containing only two cationic metals, LTHs incorporate three distinct metal cations, offering potentially superior performance in water treatment applications through their distinctive composition.^24^ Their two-dimensional structure, abundant hydroxyl groups, positive surface charge, stability, safety, and affordability have attracted significant scientific attention for applications including drug delivery, energy storage, CO_2_ adsorption, sensors, UV resistance, water treatment, photocatalytic degradation, and corrosion protection.^32–36^

Thermal treatment of layered triple hydroxides at moderate temperatures (400–600 °C) induces profound structural transformations, yielding layered triple oxides (LTOs), also referred to as mixed metal oxides.^24,37,38^ Calcination drives dehydroxylation of the brucite-type layers and elimination of interlayer anions as volatile products, causing collapse of the ordered layered architecture into poorly crystalline mixed metal oxide phases, often with spinel-type configurations. Despite structural collapse, the resulting oxides typically exhibit enhanced specific surface areas and modified porosity compared to their pristine precursors.^24^ Notably, these calcined materials retain a crystallographic “memory” of the original layered architecture, enabling reconstruction upon rehydration in aqueous environments containing appropriate anions—a phenomenon known as the memory effect. When LTO is exposed to dye solutions, water molecules re-enter the structure, hydroxide layers progressively reform, and anions from the medium are incorporated into regenerating interlayer galleries, effectively trapping pollutant molecules during reconstruction.^39,40^ The oxide surface also presents coordinatively unsaturated metal sites that participate in complexation with dye functional groups.^41,42^

Despite extensive research on layered double hydroxides for wastewater treatment, ternary systems containing three distinct metal cations remain largely unexplored, and direct comparative investigations of pristine layered triple hydroxides *versus* their calcined oxides derived from identical precursors are virtually absent. Consequently, how thermal transformation alters adsorption behavior, mechanism, and performance under realistic matrix conditions remains poorly understood. This study addresses these gaps by synthesizing a Zn–Cu–Fe LTH *via* coprecipitation and its calcined LTO counterpart. A comprehensive investigation of Indigo Carmine adsorption is undertaken, encompassing equilibrium isotherm modeling using two-parameter and three-parameter models at varying temperatures, kinetic analysis, and thermodynamic evaluation. The influence of pH, ionic strength, humic acid, and competitive dye systems is systematically examined to assess practical applicability. Post-adsorption characterization elucidates the mechanistic distinction between edge and surface-sites anion exchange in LTH and memory effect-driven reconstruction in LTO. This comparative approach establishes structure–property-performance relationships essential for designing effective adsorbents for anionic dye removal from complex aqueous matrices.

## 2 Experimental sections

### 2.1 Materials and regents

The SI provides a comprehensive description of the materials, experimental setup, and material characterization.

### 2.2 Preparation of Zn–Cu–Fe layered triple hydroxide (LTH) and derived oxide (LTO)

The Zn–Cu–Fe layered triple hydroxide (LTH) precursor was synthesized *via* a controlled co-precipitation route under constant alkaline pH conditions. Aqueous solutions of zinc acetate dihydrate (Zn(CH_3_COO)_2_·2H_2_O, ≥99.5%), copper(ii) sulfate pentahydrate (CuSO_4_·5H_2_O, ≥99.0%), and iron(iii) acetate (Fe(CH_3_COO)_3_, 97%) were prepared in deionized water (18.2 MΩ cm^−1^) and combined to yield a total cation concentration of 0.25 M. The mixed metal solution was added dropwise into a reaction vessel containing a vigorously stirred alkaline precipitating agent. This precipitating agent consisted of a 1.0 M mixed aqueous solution of sodium hydroxide (NaOH, ≥98%) and sodium carbonate (Na_2_CO_3_, ≥99.5%) in a 2 : 1 molar ratio, which served to maintain a stable pH of 10.0 ± 0.2 throughout the precipitation process while providing a carbonate interlayer source for the LTH structure. Following complete precipitation, the resulting suspension was subjected to a two-step aging protocol: first, a primary aging step at 80 °C for 12 h with continuous stirring, followed by a secondary aging step where the slurry was statically held at room temperature (25 °C) for an additional 48 h. The matured LTH precipitate was then isolated *via* vacuum filtration, washed copiously with deionized water and absolute ethanol until the filtrate reached neutrality (pH ∼7) and tested negative for sulfate ions (BaCl_2_ test), and dried at 80 °C for 12 h. The final LTO (ZnFe_2_O_4_/ZnO/CuO mixed metal oxide) was obtained through thermal decomposition. The dried LTH precursor was calcined in a programmable tube furnace under a static air atmosphere at 500 °C for 2 h, employing a controlled heating ramp of 3 °C min^−1^. The detailed synthesis procedure and basic characterization of the LTO sample were reported in our previous work.^43^

## 3 Results and discussion

### 3.1 Characterization of LTH and LTO

**Fig. 1:**
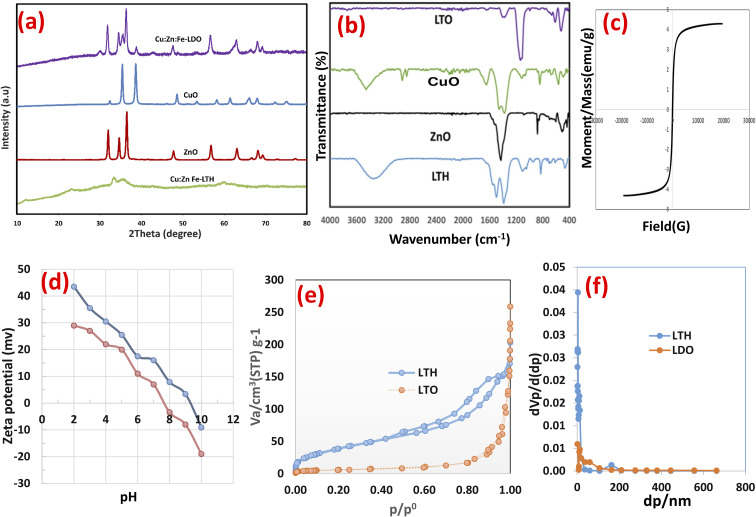
Characterization of the synthesized samples (a) XRD, (b) FTIR, (c) magnetization, (d) zeta potential (e) N_2_-adsorption–desorption isotherm, and (f) pore size distribution of LTH and LTO.

The crystalline structures of the synthesized Zn–Cu–Fe LTH and its calcined LTO were examined by powder X-ray diffraction (Fig. 1a). The pristine LTH pattern exhibits reflections characteristic of a hydrotalcite-like layered hydroxide structure, although the peaks are relatively broad, asymmetric, and of low to moderate intensity, together with a noticeable amorphous background contribution.^44,45^ These features likely arise from the incorporation of multiple transition metals (Zn^2+^, Cu^2+^, and Fe^3+^) into the hydroxide layers, where differences in ionic radii and the Jahn–Teller distortion associated with Cu^2+^ induce structural disorder and partial amorphization. Nevertheless, the main basal reflection observed at 2*θ* = 11.8° corresponds to the (003) plane, giving an interlayer *d*-spacing of approximately 7.5 Å according to Bragg's law, consistent with carbonate-intercalated layered hydroxides (ICDD 00-048-0083 used as a structural reference). Additional broader reflections centered near 23.6° (006), 34.6° (012), and 60.5° (110) further support the layered stacking structure. The (110) reflection gives a metal–metal distance (*a* = 2 × *d*_110_) of approximately 3.06 Å. No distinct reflections attributable to separate crystalline metal hydroxide or oxide impurities were observed, supporting the formation of a single layered phase despite the partial amorphous character.

After calcination at 500 °C, the layered reflections disappear completely, indicating dehydroxylation and removal of interlayer carbonate species.^44^ The resulting LTO pattern consists of a heterogeneous mixture of crystalline oxide phases. Reflections at 31.92°, 34.58°, and 36.41° are assigned to hexagonal ZnO (zincite, ICDD 01-080-0074), corresponding to the (100), (002), and (101) planes, respectively. Peaks at 35.56° and 38.82° correspond to monoclinic CuO (tenorite, ICDD 00-048-1548) indexed to the (002) and (111) planes. Additional reflections at 30.08°, 35.56° (overlapping with CuO), 42.96°, 56.68°, and 62.90° are attributed to the spinel ZnFe_2_O_4_ phase (ICDD 00-077-0011), indexed to the (220), (311), (400), (511), and (440) planes.^43^ The broader full width at half maximum values of the LTO reflections suggest relatively small crystallite sizes resulting from the thermal transformation process. Overall, calcination converts the partially crystalline layered precursor into a heterogeneous nanocomposite composed of intimately mixed ZnO, CuO, and ZnFe_2_O_4_ phases.

The FTIR spectrum of pristine LTH reveals absorption bands characteristic of hydrotalcite-like layered materials (Fig. 1b). A broad band centered at 3300 cm^−1^ arises from O–H stretching of hydroxyl groups and interlayer water molecules, with its width indicating extensive hydrogen bonding.^46^ Weak bands at 2928 and 2856 cm^−1^ correspond to asymmetric and symmetric stretching of interlayer carbonate anions, while the band at 2360 cm^−1^ is attributed to adsorbed atmospheric CO_2_.^45^ The bending vibration of interlayer water appears at 1544 cm^−1^. Below 800 cm^−1^, bands associated with metal–oxygen and metal–hydroxyl lattice vibrations confirm formation of the layered framework, encompassing Zn–O, Cu–O, and Fe–O stretching modes within octahedral sheets.^46^

Thermal treatment at 500 °C induces marked changes in the LTO spectrum. The broad O–H stretching band diminishes substantially, reflecting extensive dehydroxylation of the brucite-like layers. Complete disappearance of the 1544 cm^−1^ bending vibration confirms removal of interlayer water, while attenuation of carbonate-related bands indicates thermal decomposition and CO_2_ release.^43^ The low wavenumber region undergoes significant transformation, with new absorption bands emerging from metal–oxygen vibrations in the newly formed oxide phases. These bands, broader and less defined than those in LTH, suggest reduced crystallinity and increased structural disorder—consistent with XRD observations. Residual O–H stretching persists, likely from partial rehydroxylation upon ambient exposure or incomplete dehydroxylation at the calcination temperature.

The magnetic behavior of LTO was investigated by vibrating sample magnetometry at room temperature (Fig. 1c). Unlike the pristine LTH precursor—which exhibits no magnetic response due to paramagnetic Fe^3+^ ions within a diamagnetic layered matrix—the calcined sample displays clear ferromagnetic behavior. The magnetization curve reveals a distinct hysteresis loop. As the applied field increases to 30 000 Oe, magnetization rises rapidly and approaches saturation near 5 emu per g.^43^ Upon field reversal, remanent magnetization of approximately 2 emu per g remains at zero field, with coercivity determined at around 5000 Oe. This magnetic ordering arises from structural transformation during calcination, where non-magnetic LTH decomposes into a heterogeneous oxide mixture including CuO, ZnO, and iron-rich spinel phases such as ZnFe_2_O_4_. These spinel ferrites exhibit ferrimagnetic ordering from antiparallel moment alignment on tetrahedral and octahedral sites. While modest compared to pure magnetite, the observed saturation magnetization confirms successful formation of magnetic phases. Practically, this acquired magnetism enables facile separation of spent LTO from solution using an external magnetic field—a distinct advantage over non-magnetic LTH that eliminates centrifugation or filtration steps, reducing operational costs and processing time.

The surface charge characteristics of LTH and LTO were evaluated across pH 2.0–10.0 (Fig. 1d). LTH exhibits strong pH-dependent behavior, with zeta potential decreasing from +43.5 mV at pH 2 to −9.1 mV at pH 10.^46^ The point of zero charge (pH_pzc_) occurs at approximately pH 8.2. This progressive charge reversal reflects protonation and deprotonation of surface hydroxyl groups (M–OH) on the layered sheets.^47^ The high positive charge at acidic pH arises from abundant –OH_2_^+^ species on the hydroxyl-rich LTH surface, while deprotonation to M–O^−^ dominates under alkaline conditions. LTO follows a similar trend but with systematically lower values: +29.0 mV at pH 2 to −19.0 mV at pH 10, with pH_pzc_ shifted to approximately 7.5. This downward shift indicates fundamental changes in surface chemistry following calcination. Thermal transformation from hydroxide to oxide phases reduces surface hydroxyl density and alters their acid-base character. The oxide surface presents coordinatively unsaturated metal sites that behave as Lewis acids, while remaining hydroxyl groups exhibit greater acidity due to electron-withdrawing effects from the oxide lattice. Consequently, LTO deprotonates more readily, explaining the lower pH_pzc_. The presence of multiple metal centers—Zn^2+^, Cu^2+^, and Fe^3+^—in both materials creates heterogeneous surface sites with varying proton affinities. In LTO, formation of spinel phases (ZnFe_2_O_4_/CuFe_2_O_4_) introduces additional surface complexity, as oxygen atoms bonded to different cations exhibit distinct basicity. At near-neutral pH relevant for wastewater treatment (pH 6–7), LTH retains substantial positive charge (+17.5 to +16 mV) favorable for electrostatic attraction of anionic Indigo Carmine, while LTO approaches charge neutrality (+11 to +7 mV). This difference confirms that electrostatic interactions contribute more significantly to dye uptake onto pristine LTH, whereas LTO relies increasingly on alternative mechanisms—surface complexation or memory effect-driven reconstruction—consistent with its reduced but still substantial adsorption capacity.

The textural characteristics of LTH and LTO were investigated through nitrogen adsorption–desorption measurements at 77 K, with the isotherms and corresponding pore size distributions presented in Fig. 1e and f. LTH exhibits a Type IV isotherm according to IUPAC classification, characteristic of mesoporous materials.^48,49^ A distinct hysteresis loop appears in the relative pressure range *p*/*p*^0^ = 0.4–1.0, classified as H3-type, which is typically associated with slit-shaped pores formed by aggregation of plate-like particles—consistent with the layered morphology of LTH. The adsorption branch rises gradually at low relative pressures (*p*/*p*^0^ < 0.4) due to monolayer-multilayer adsorption on mesopore walls, followed by a steep increase at higher pressures corresponding to capillary condensation within mesopores. The BET surface area of LTH is calculated as 150.14 m^2^ g^−1^, with total pore volume of 0.2645 cm^3^ g^−1^ at *p*/*p*^0^ = 0.990. The mean pore diameter of 7.05 nm confirms the mesoporous nature, with pore sizes predominantly in the small mesopore range favorable for dye molecule diffusion and access to active sites.

Following calcination, LTO exhibits a markedly different textural profile. The isotherm remains Type IV but with significantly reduced nitrogen uptake across all relative pressures, reflected in the lower BET surface area of 21.70 m^2^ g^−1^—an approximately seven-fold decrease from LTH. The hysteresis loop shifts to higher relative pressures (*p*/*p*^0^ = 0.6–1.0) and becomes less pronounced, indicating altered pore architecture. Total pore volume decreases to 0.1982 cm^3^ g^−1^, while mean pore diameter expands substantially to 36.53 nm, placing LTO in the large mesopore to small macropore range. This pore widening accompanies structural collapse during calcination, where dehydroxylation and elimination of interlayer carbonate cause layer stacking disruption and coalescence of smaller pores into larger voids. The pore size distribution derived from the desorption branch using the BJH method confirms this evolution: LTH displays a narrow distribution centered around 3–5 nm, while LTO shows broader distribution with pores extending beyond 30 nm.^43^ The reduced surface area and enlarged pores of LTO explain its lower adsorption capacity compared to LTH, as fewer active sites are available per unit mass despite potentially improved pore accessibility for larger dye aggregates. However, the preserved mesoporosity ensures that diffusion limitations do not severely impede dye uptake, while the altered pore network may facilitate different adsorption mechanisms including pore filling and surface complexation on exposed oxide surfaces.

**Fig. 2:**
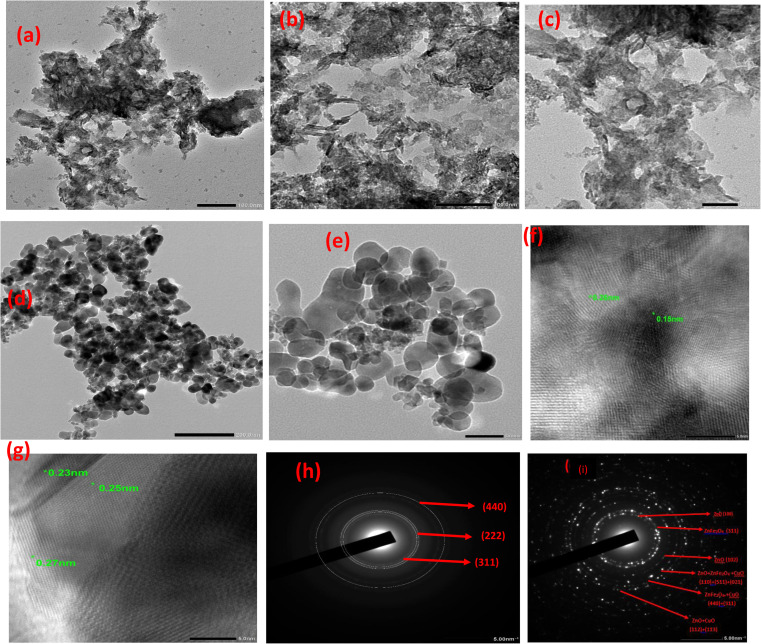
TEM micrographs of (a–c) LTH and (d and e) LTO; HRTEM images of (f) LTH and (g) LTO; SAED patterns of (h) LTH and (i) LTO.

The TEM image of pristine LTH (Fig. 2a–c) reveals well-defined two-dimensional hexagonal plate-like morphology characteristic of layered double hydroxides. The hexagonal plates exhibit uniform contrast with lateral dimensions ranging from approximately 100–200 nm and relatively thin thickness, indicating successful formation of the layered structure with good crystallinity. The plates display smooth surfaces and sharp edges, reflecting well-ordered layer stacking without significant amorphous coating. In contrast, the TEM image of LTO (Fig. 2d–e) shows complete morphological transformation following calcination at 500 °C. The uniform hexagonal plates of LTH entirely disappear, replaced by a heterogeneous mixture of particle morphologies. Three distinct features are observed: aggregated nanoparticles approximately 20–50 nm in size forming porous clusters, irregular plate-like fragments with rough surfaces and fractured edges, and larger blocky particles resulting from sintering during thermal treatment. This morphological diversity reflects the complex oxide composition revealed by XRD, with different phases adopting characteristic shapes—ZnO often forming elongated particles, CuO appearing as irregular aggregates, and spinel ferrites presenting as equiaxed nanocrystals. The loss of the well-defined hexagonal habit confirms complete structural collapse of the layered architecture upon calcination.

HRTEM analysis was employed to elucidate the atomic-scale architecture and phase transition from the precursor to the calcined composite. For the pristine LTH, the micrographs reveal continuous and well-resolved lattice fringes extending across the crystalline domains (Fig. 2f), signifying high internal crystallinity. Measured interplanar spacings of 0.26 nm and 0.15 nm correspond precisely to the (311) and (440) planes of the LTH framework, aligning with the XRD reflections at 2theta = 34.42° and 60.62°, respectively. In contrast, the LTO composite (Fig. 2g) exhibits a complex, multi-phasic pattern where distinct lattice fringes coexist in close proximity, confirming the intimate integration of the oxide phases. Specifically, fringes at 0.28 nm are attributed to the ZnO (100) plane, while the 0.25 nm spacing reflects overlapping contributions from the ZnFe_2_O_4_ (311) and CuO (002) planes. A further fringe at 0.23 nm confirms the presence of CuO (111), establishing that the LTO is an intimately mixed ternary heterojunction rather than a collection of isolated single-phase particles.

**Fig. 3:**
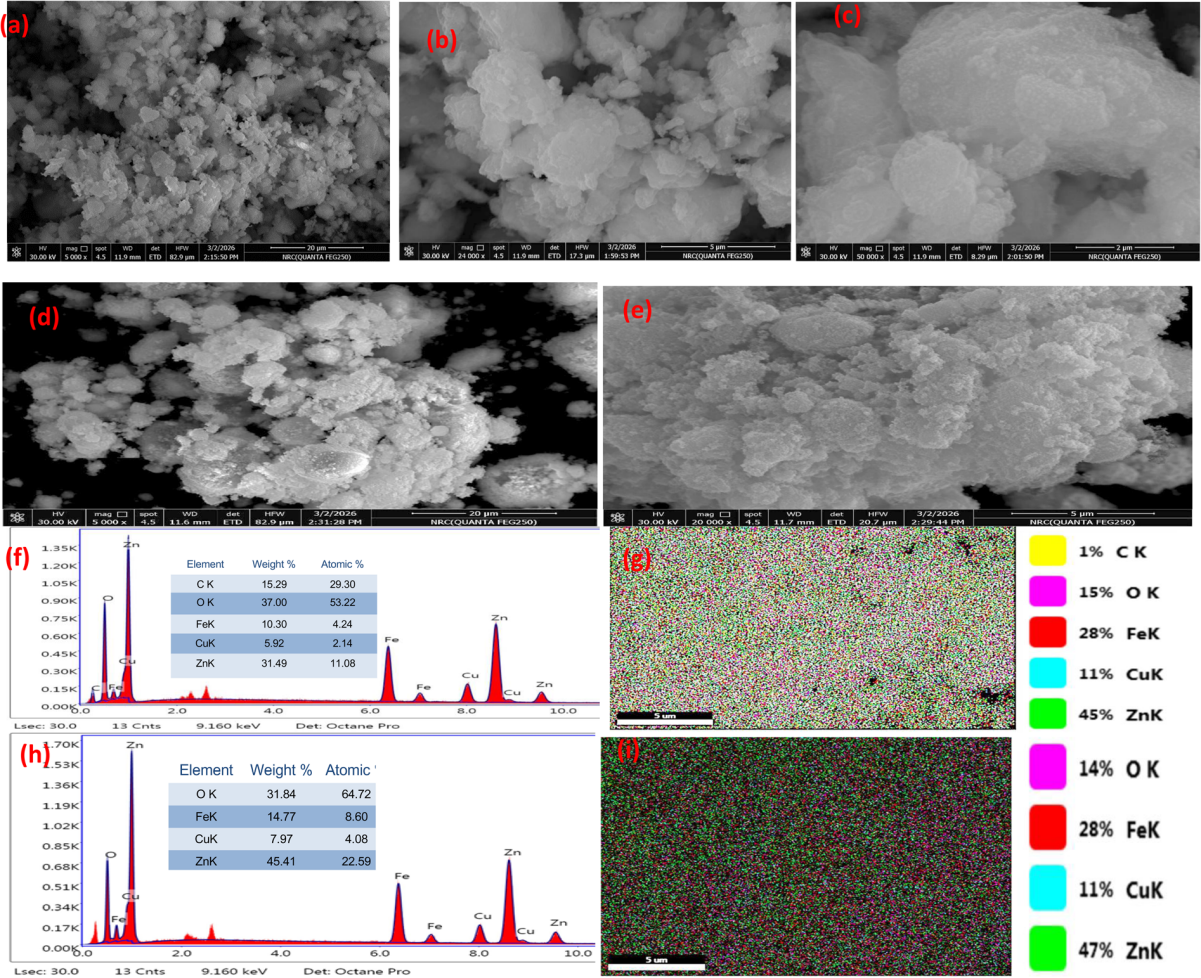
Morphological and elemental characterization of the synthesized materials: FESEM micrographs of (a–c) pristine LTH and (d and e) calcined LTO; EDS spectra and corresponding elemental mapping for (f and g) LTH and (h and i) LTO.

These findings are further substantiated by SAED patterns. The LTH pattern (Fig. 2h) displays sharp, concentric diffraction rings indexed to the (311), (222), and (440) planes, consistent with a highly ordered polycrystalline layered structure. Conversely, the LTO pattern (Fig. 3i) reveals more diffuse, continuous rings, indicating a reduction in crystallite size and increased structural disorder following the topotactic transformation. Six distinct rings were identified, representing ZnO (100, 102, 112), ZnFe_2_O_4_ (311, 440, 511), and CuO (021, 311, 113) phases. The significant overlap in rings at 0.162 nm, 0.148 nm, and 0.137 nm definitively proves the formation of a heterogeneous nanocomposite where the constituent nanocrystallites are structurally interwoven, a critical factor in enhancing the material's surface complexation and matrix resilience during dye sequestration.

The FESEM micrographs of pristine LTH (Fig. 3a–c) reveal well-defined plate-like aggregates with lateral dimensions ranging from approximately 100 to 300 nm.^47^ These plates exhibit smooth surfaces and distinct hexagonal outlines, reflecting the inherent symmetry of the layered triple hydroxide structure. The plates are densely packed with random orientations, forming stacked assemblies with occasional interparticle voids. Some larger aggregates result from face-to-face and edge-to-face associations between adjacent plates. This morphology arises from anisotropic crystal growth, where preferential lateral expansion occurs along the *a* and *b* plane while stacking proceeds along the *c*-axis through interlayer carbonate-mediated assembly.

Following calcination at 500 °C, LTO (Fig. 3d and e) displays complete morphological transformation. The smooth hexagonal plates disappear entirely, replaced by a porous network of irregularly shaped nanoparticles ranging from 20 to 80 nm. These nanoparticles exhibit rough surfaces and significant interparticle fusion through sintering, forming interconnected clusters with open porosity throughout the sample. The transformation results from simultaneous dehydroxylation and carbonate elimination, which collapse the layered framework, followed by recrystallization into mixed oxide phases—ZnO, CuO, and spinel ferrites. The resulting porous architecture, though structurally distinct from the layered precursor, provides accessible pathways for dye diffusion to surface active sites.

EDS and mapping (Fig. 3f–i) highlight compositional differences between the two materials. For LTH, carbon detection confirms interlayer carbonate balancing the positively charged layers. Zinc, copper, and iron appear in ratios close to nominal synthesis. Mapping shows all three metals co-localize uniformly across the plate-like aggregates—no phase segregation. This confirms a genuine ternary layered phase, not a physical mixture of separate hydroxides.

After calcination, carbon disappears entirely (carbonate loss), oxygen signals drop (dehydroxylation), and metal signals intensify from concentration of non-volatile oxides. Mapping becomes more complex: zinc remains widely dispersed, while copper and iron show areas of relative enrichment. Some co-localization persists, but partial phase separation is evident. Iron-enriched zones with associated zinc correspond to ZnFe_2_O_4_ spinel domains, while copper-only areas represent CuO crystallites and zinc-only areas represent ZnO. LTO is therefore a heterogeneous composite of intimately mixed ZnO, CuO, and ZnFe_2_O_4_ phases—each offering different surface chemistry for dye interaction, unlike the uniform layered surface of LTH.

### 3.2 Adsorption studies

#### 3.2.1 Batch adsorption

##### 3.2.1.1 Effect of contact time on Indigo Carmine adsorption

**Fig. 4:**
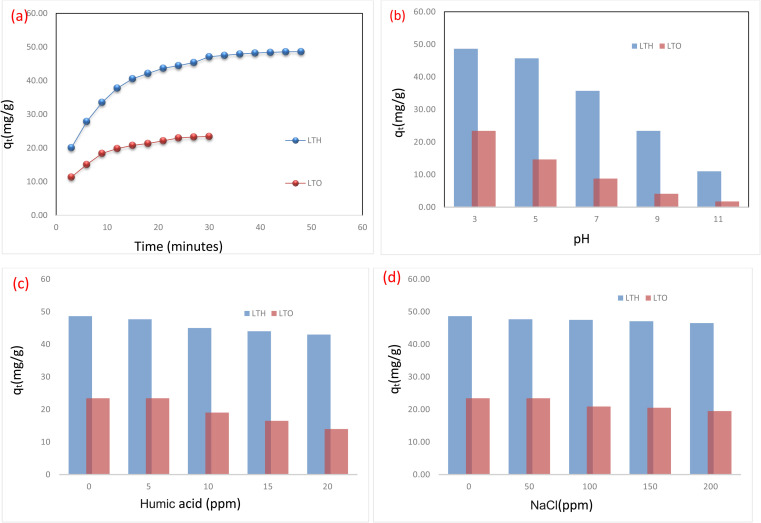
Batch adsorption: (a) effect of contact time, (b) effect of pH, (c) effect of humic acid, and (d) effect of NaCl.

The removal efficiency of Indigo Carmine by both adsorbents as a function of time is presented in Fig. 4a. LTH exhibits rapid initial uptake—within the first 3 min, removal reaches 34.3%, increasing to 47.6% at 6 min and 57.3% at 9 min. This fast kinetics reflects the high surface area (150.14 m^2^ g^−1^) and abundant positively charged sites (+17.5 mV at pH 6) readily accessible to anionic dye molecules.^44^ The well-developed hexagonal plate morphology with exposed edges facilitates immediate electrostatic attraction and anion exchange of edges and surface sites by IC molecules.^47^ After 15 min, removal slows progressively as surface sites become occupied: 69.3% at 15 min, 72.0% at 18 min, 74.6% at 21 min. Equilibrium approaches gradually beyond 30 min, reaching 80.4% at 30 min and slowly increasing to 83% at 48 min. This slight continued increase suggests slow diffusion into less accessible interlayer regions or reorganization of adsorbed molecules within the galleries.

LTO displays distinctly different behavior. Initial uptake is notably slower—19.4% at 3 min compared to 34.3% for LTH. This cannot be attributed to pore diffusion limitations, as LTO possesses larger pores (36.53 nm) than LTH (7.05 nm). Rather, the slower kinetics arises from three interconnected factors. First, surface area is substantially lower (21.70 m^2^ g^−1^), providing fewer immediate binding sites. Second, the adsorption mechanism differs fundamentally. Unlike LTH where rapid anion exchange dominates, LTO relies on the memory effect—gradual reconstruction of the layered structure upon rehydration, incorporating dye anions into reforming interlayer galleries. This reconstruction is time-dependent, requiring progressive uptake of water and anions. Third, the heterogeneous surface comprising ZnO, CuO, and ZnFe_2_O_4_ phases offers diverse binding sites through surface complexation on coordinatively unsaturated metal sites and residual electrostatic interactions (+11 mV at pH 6). Removal reaches 25.8% at 6 min, 31.4% at 9 min, then increases more slowly—33.9% at 12 min, 35.5% at 15 min, 36.5% at 18 min, 37.8% at 21 min, 39.3% at 24 min, reaching 40% at 30 min where equilibrium occurs. The absence of further increase after 30 min confirms that reconstruction completes within this timeframe at this concentration. LTO thus reaches equilibrium earlier and at lower capacity (40% at 30 min) compared to LTH (83% at 48 min). This reflects its reduced surface area and fundamentally different adsorption mechanism—involving time-dependent memory effect reconstruction coupled with surface complexation on oxide phases—rather than the rapid surface and edge-sites anion exchange dominating in pristine LTH.

#### 3.2.2 Effect of pH

The removal of Indigo Carmine by both adsorbents shows strong dependence on solution pH (Fig. 4b), with trends directly reflecting their surface charge characteristics.^49^ For LTH, maximum uptake occurs at acidic conditions and progressively declines as pH increases. This behavior arises from electrostatic interactions between anionic dye molecules and the variable surface charge of the layered material.^48^ At low pH, extensive protonation of surface hydroxyl groups generates strong positive charge on the LTH sheets, creating powerful electrostatic attraction toward negatively charged IC molecules.^47^ As pH approaches the point of zero charge near pH 8, surface positive charge diminishes, weakening electrostatic attraction. Beyond the pH_pzc_, the surface becomes negatively charged, causing electrostatic repulsion that suppresses adsorption almost completely.^46^

LTO exhibits similar pH-dependent behavior but with consistently lower uptake across all conditions. Maximum removal occurs at acidic pH where the oxide surface carries positive charge from protonated metal–OH groups. However, the capacity remains substantially below LTH even at low pH—reflecting its lower surface area and fewer available binding sites rather than differences in electrostatic attraction alone. As pH increases toward the LTO point of zero charge near pH 7.5, uptake declines. Importantly, significant adsorption persists in the near-neutral pH range despite diminished positive charge. This indicates that mechanisms beyond electrostatics contribute: surface complexation on coordinatively unsaturated metal sites of ZnO, CuO, and ZnFe_2_O_4_ phases, as well as memory effect reconstruction that can proceed even under near-neutral conditions. At alkaline pH, the strongly negative oxide surface repels anionic dye, while competition from excess OH^−^ ions for reconstruction sites further suppresses uptake.

#### 3.2.3 Effect of humic acid

The presence of co-existing substances in real wastewater—specifically natural organic matter represented by humic acid and inorganic salts represented by sodium chloride—exerts distinct and revealing effects on the adsorption performance of LTH and LTO (Fig. 4c). For humic acid, a clear divergence emerges between the two materials. LTH demonstrates remarkable resilience, retaining the vast majority of its Indigo Carmine uptake capacity even at the highest humic acid concentration tested. This tolerance arises because LTH adsorbs IC exclusively through electrostatic attraction to its positively charged external surfaces and edge sites; humic acid, despite being polyanionic and capable of competing for these same sites, cannot fully displace IC due to the high surface charge density and the relatively rapid kinetics of IC binding. In contrast, LTO suffers a much more pronounced decline in capacity with increasing humic acid concentration. The reason lies in LTO's multimodal adsorption mechanism: humic acid molecules—bearing numerous carboxylic and phenolic functional groups—can strongly coordinate to the same coordinatively unsaturated metal centers (Zn^2+^, Cu^2+^, Fe^3+^) on the ZnO, CuO, and ZnFe_2_O_4_ phases that are also responsible for IC complexation. Additionally, the large, flexible structure of humic acid may physically coat the oxide surface or interfere with the memory effect reconstruction process, effectively blocking access to multiple uptake pathways simultaneously. The practical implication is that for wastewater streams rich in natural organic matter—such as municipal wastewater or effluents from biological treatment—LTH is clearly the more robust choice.

#### 3.2.4 Effect of NaCl

Turning to sodium chloride, both materials exhibit progressive inhibition of IC adsorption with increasing ionic strength, but again with important differences (Fig. 4d). The inhibitory effect stems from two complementary mechanisms: direct competition from chloride anions for positively charged binding sites, and compression of the electrical double layer which reduces the electrostatic attraction between the anionic IC molecule and the adsorbent surface. LTH, with its higher point of zero charge and greater surface charge density, maintains strong electrostatic attraction even at elevated salt concentrations, losing only a modest fraction of its capacity at the highest NaCl level tested. The layered structure's high density of hydroxyl groups and interlayer carbonate anions at the edges and surface-sites provide sufficient binding energy that chloride ions cannot fully outcompete IC. LTO, however, is more sensitive to ionic strength increases. Its lower surface charge means that even minor salt addition significantly weakens the already-modest electrostatic contribution to IC uptake. Furthermore, chloride ions may adsorb onto the oxide surface, passivating the coordinatively unsaturated metal centers that would otherwise participate in surface complexation with IC. The memory effect, requiring specific anion incorporation during layer reconstruction, may also be disrupted in chloride-rich environments, as chloride competes with IC for inclusion into reforming interlayer galleries. The net result is that LTO loses a substantially larger proportion of its capacity at high NaCl concentrations compared to LTH.

The key takeaway is that both LTH and LTO are robust adsorbents capable of performing well under realistic matrix conditions. LTH emerges as the more resilient option, retaining over 85% of its original capacity even under the most challenging interference levels, making it ideal for complex wastewaters rich in organic matter or salts. LTO, while somewhat more sensitive, still delivers effective IC removal and retains its magnetic separability advantage, remaining a viable choice for applications where rapid recovery is prioritized over maximum interference tolerance. The practical message for wastewater engineers is clear: both materials are suitable for real-world use, and the choice between them should be guided by the specific matrix composition and operational priorities rather than by concerns about performance collapse under interference.

#### 3.2.5 Kinetic and adsorption isotherms models and thermodynamic studies

##### 3.2.5.1 PFO and PSO models

**Fig. 5:**
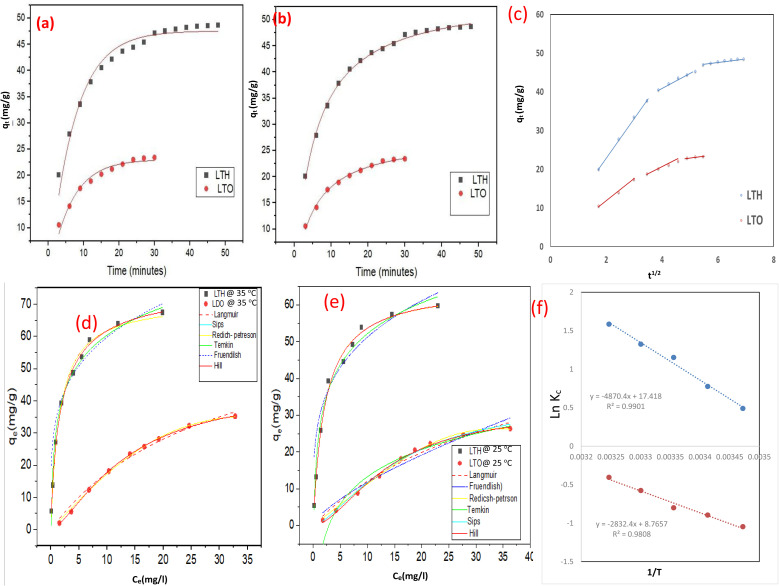
(a)Non-linear kinetic plots for adsorption of IC dye on LTH and LTO: (a) pseudo first-order, (b) pseudo second-order, and (c) intraparticle diffusion plot, (d and e) nonlinear adsorption isotherms at different temperatures, (f) Arrhenius plots.

The kinetics of IC adsorption onto both materials were evaluated using non-linear pseudo-first-order (PFO) and pseudo-second-order (PSO) models, with fitted parameters presented in Table 1 and corresponding plots in Fig. 5a. For LTH, the experimental equilibrium capacity (*q*_exp_) is 48.6 mg g^−1^. The PFO model calculates *q*_calc_ of 47.6 mg g^−1^ with rate constant *k*_1_ of 0.139 min^−1^ and *R*^2^ of 0.97. The PSO model yields *q*_calc_ of 54.9 mg g^−1^ with *k*_2_ of 0.00331 g mg^−1^ min^−1^ and *R*^2^ of 0.99.^44^ Although both models show reasonable correlation, the closer agreement between *q*_exp_ and PFO qcalc (47.6 *vs.* 48.6 mg g^−1^) indicates that PFO better describes LTH kinetics. This is consistent with physisorption-dominated processes where the rate depends on available external surface sites—typical for electrostatic attraction of IC to the positively charged LTH surface and edges.

Kinetics model parameters

**Table:** 

Model	IC dye	
	LTH	LTO
**Pseudo first order**		
*q*_exp_ (mg g^−1^)	48.6	23.4
*q*_Calc_ (mg g^−1^)	47.59	23.2
*K*_1_ (min^−1^)	0.1392	0.1616
*R* ^2^	0.97	0.967

**Pseudo second order**		
*q*_exp (_mg g^−1^)	48.6	23.4
*q*_Calc_ (mg g^−1^)	54.9	27.7
*K*_2_(g mg^−1^ min^−1^)	0.00331	0.0067
*R* ^2^	0.99	0.99

**Table:** 

Weber–Morris			
First stage	*K* (mg g^−1^ min^−0.5^)	10.6	5.5
	*C*	2.52	1.7
Second stage	*K* (mg g^−1^ min^−0.5^)	3.26	2.019
	*C*	27.6	12.87
Third stage	*K* (mg g^−1^ min^−0.5^)	1.06	0.73
	*C*	41.4	19.42

For LTO, *q*_exp_ is 23.4 mg g^−1^. The PFO gives *q*_calc_ of 23.2 mg g^−1^ with *k*_1_ of 0.162 min^−1^ and *R*^2^ of 0.967. The PSO calculates *q*_calc_ of 27.7 mg g^−1^ with *k*_2_ of 0.0067 g mg^−1^ min^−1^ and *R*^2^ of 0.99. The closer match between *q*_exp_ and PFO *q*_calc_ (23.2 *vs.* 23.4 mg g^−1^) again favors PFO, despite the slightly higher *R*^2^ for PSO. The rate constant *k*_1_ for LTO (0.162 min^−1^) exceeds that of LTH (0.139 min^−1^), indicating faster initial uptake per available site. However, this does not translate to higher capacity because fewer total sites exist—reflected in the lower *q*_exp_ and the lower pre-exponential factor (23.0 *vs.* 47.5 in the PFO equation). The faster rate constant for LTO likely reflects the absence of diffusion limitations (larger pores, 36.5 nm) compared to LTH (7.0 nm), allowing rapid access to available surface sites. However, once those limited sites are occupied, no further uptake occurs.

The PSO model consistently overestimates equilibrium capacities for both materials—more severely for LTO (27.7 *vs.* 23.4 mg g^−1^) than LTH (54.9 *vs.* 48.6 mg g^−1^). This indicates that chemisorption as the sole rate-controlling step does not fully describe either system. For LTH, the mechanism combines rapid electrostatic attraction to external surfaces and edge sites, with possible partial intercalation at crystallite margins where layer expansion accommodates dye molecules. For LTO, the mechanism involves time-dependent memory effect reconstruction coupled with surface complexation on ZnO, CuO, and ZnFe_2_O_4_ phases—processes that PFO captures better by accounting for gradually diminishing available sites. The kinetic analysis thus confirms that physisorption-related mechanisms dominate both systems, with LTH relying on electrostatic attraction to external layered surfaces and LTO on reconstruction-mediated and complexation uptake on mixed oxide phases.

##### 3.2.5.2 Intra-particle diffusion model

The Weber–Morris model was applied to identify rate-controlling steps during Indigo Carmine adsorption, with parameters summarized in Table 1. The multi-linear plots for both adsorbents indicate that multiple processes govern the overall adsorption rate, each reflecting different dye transfer mechanisms (Fig. 5c).^45^

For LTH, three distinct stages appear. The first sharp linear segment represents instantaneous external surface binding—dye molecules transfer from bulk solution to the external surface through film diffusion and rapidly occupy readily accessible sites *via* electrostatic attraction.^45^ The high rate constant reflects abundant positively charged sites and the absence of diffusion barriers at this initial phase. The non-zero intercept confirms that boundary layer resistance exists even at this stage, indicating that film diffusion contributes alongside surface binding. The second stage corresponds to gradual intra-particle diffusion, where dye molecules migrate into interparticle galleries and smaller mesopores. The reduced rate constant reflects slower transport through narrower pathways and increasing diffusion path lengths. The higher intercept indicates thickening of the boundary layer as adsorption proceeds. The third stage represents final equilibrium, where intra-particle diffusion slows considerably as active sites saturate and residual dye concentration diminishes—adsorption becomes limited by availability rather than transport.

LTO exhibits similar three-stage behavior but with distinct characteristics. The first stage shows a lower rate constant, reflecting fewer immediately accessible surface sites due to reduced surface area. Despite larger pores, intra-particle diffusion rates in second and third stages remain lower than LTH—confirming that the adsorption mechanism itself, not pore diffusion, controls kinetics. The memory effect requires gradual structural reorganization and anion incorporation, processes inherently slower than simple pore diffusion. Lower intercept values throughout suggest thinner boundary layers, possibly due to the rough, heterogeneous surface of aggregated oxide nanoparticles disrupting uniform film formation compared to smooth LTH plates.

The decreasing rate constants from first to third stages for both materials reflect progressively slower uptake as external sites saturate and diffusion pathways lengthen. The absence of origin-passing segments confirms that intra-particle diffusion is not the sole rate-controlling step—external mass transfer (film diffusion) contributes significantly throughout. This multi-step control is consistent with PFO kinetics and confirms complex adsorption mechanisms combining film diffusion, intra-particle transport, and for LTO, time-dependent structural reconstruction.

##### 3.2.5.3 Adsorption isotherms

Equilibrium adsorption data for Indigo Carmine onto both materials were evaluated using Langmuir, Freundlich, Sips, Redlich–Peterson, Temkin, and Hill models at 25 °C and 35 °C, with fitted parameters presented in Table 2. The comparative analysis reveals fundamentally different adsorption behaviors reflecting the distinct structural characteristics of each material (Fig. 5d and e).^46,47^

**Table 2:** Adsorption isotherms parameters

Model	LTH		LTO	
	25 °C	35 °C	25 °C	35 °C
**Langmuir model**				
*q*_max_ (mg g^−1^)	65.29 ± 1.43	71.34 ± 1.42	56.53 ± 10.08	69.15 ± 7.4
*b* (L mg^−1^)	0.463 ± 0.03	0.638 ± 0.050	0.0271 ± 0.0078	0.0342 ± 0.006
Reduced Chi^2^	2.36	2.609	2.097	1.577
*R* ^2^	0.99	0.99	0.97	0.98

**Freundlich model**				
*K*_f_	28.54 ± 0.21	34.83 ± 0.24	2.30 ± 0.62	3.88 ± 0.03
n	3.93 ± 0.04	4.27 ± 0.05	1.41 ± 0.17	1.53 ± 0.005
Reduced Chi^2^	8.76	11.74	4.479	0.5366
*R* ^2^	0.92	0.90	0.95	0.99

**Temkin isotherm**				
*B*	12.12 ± 0.06	12.54 ± 0.61	10.68 ± 0.06	11.58 ± 0.09
*K*_*T* (L mg^−1^)	0.41 ± 0.16	12.30 ± 2.23	0.346 ± 0.004	0.570 ± 0.009
*b* (J moL^−1^)	204.52	204.28	232.13	221.33
Reduced Chi^2^	2.6	9.08	1.88	4.938
*R* ^2^	0.976	0.98	0.96	0.94

**Sips isotherm**				
*q*_max_ (mg g^−1^)	65.48 ± 3.00	76.71 ± 2.60	35.56 ± 0.23	44.71 ± 1.08
*b* (L mg^−1^)	0.460 ± 0.06	0.528 ± 0.056	0.0825 ± 0.0006	0.0752 ± 0.0029
*n* (heterogeneity factor)	0.993 ± 0.09	0.851 ± 0.050	1.508 ± 0.012	1.467 ± 0.043
Reduced Chi^2^	2.75	1.308	0.249	0.0733
*R* ^2^	0.99	0.99	0.99	0.99

**Redlich–Peterson isotherm**				
*b* (g)	0.985 ± 0.04	0.92 ± 0.02	3.1 ± 0.67	2.08518 ± 0.32
*a* (L mg^−1^)^*β*^	0.49 ± 0.13	0.95 ± 0.15	7.6*10^−6^ ± 1.9*10^−5^	5.2*10^−4^ ± 6.4*10^−4^
*K* (L g^−1^)	31.24 ± 4.18	55.9 ± 0	1.14 ± 0.04	1.832 ± 0.079
*q*_max_ (mg g^−1^)	31.8	60.7	0.36	0.86
Reduced Chi^2^	2.7	1.38	0.36	0.37
*R* ^2^	0.99	0.99	0.99	0.99

**Hill**				
*q*_max_ (mg g^−1^)	65.47 ± 2.99	76.711 ± 2.5	31.7 ± 1.6	44.711 ± 1.07
*K*_H_(mg L^−1^)	2.17 ± 0.28	1.895 ± 0.19	13.7 ± 0.99	13.29 ± 0.5
*n*	0.993 ± 0.09	0.850 ± 0.049	1.74 ± 0.136	1.467 ± 0.042
Reduced Chi^2^	2.275	1.308	0.32	0.07334
*R* ^2^	0.99	0.99	0.99	0.99

For LTH, the Langmuir model provides excellent fit at both temperatures (*R*^2^ = 0.99) with maximum capacities increasing from 65.29 mg g^−1^ at 25 °C to 71.34 mg g^−1^ at 35 °C. This temperature-dependent increase indicates endothermic adsorption.^46^ The Sips model shows nearly identical *q*_0_ values (65.48 mg g^−1^ at 25 °C, 76.71 mg g^−1^ at 35 °C) with heterogeneity factors of 0.993 and 0.851—very close to unity—confirming that LTH behaves essentially as a homogeneous surface. The Hill model yields similar parameters with *n* values near 1, indicating non-cooperative adsorption. Redlich–Peterson *g* values of 0.985 and 0.92 approach unity, further supporting Langmuir-type behavior. The Freundlich model shows poorer fit (*R*^2^ = 0.92–0.90) with *n* values of 3.93–4.27, indicating that multilayer adsorption is not dominant. Temkin constants (*B* = 12.12–12.54) suggest moderate adsorbate–adsorbent interactions. The consistent agreement between Langmuir, Sips (with *n* ≈ 1), and Redlich–Peterson (*g* ≈ 1) conclusively demonstrates that Indigo Carmine adsorption onto LTH proceeds *via* monolayer coverage on a homogeneous surface, with adsorption sites uniformly distributed throughout the layered structure—consistent with its well-defined hexagonal plates and uniform interlayer galleries revealed by XRD and HRTEM.

For LTO, the adsorption behavior differs markedly. The Sips model provides the best fit at both temperatures (*R*^2^ = 0.99) with heterogeneity factors of 1.508 at 25 °C and 1.467 at 35°C—significantly greater than unity—confirming surface heterogeneity and cooperative adsorption. Maximum capacities from Sips increase from 35.56 mg g^−1^ at 25 °C to 44.71 mg g^−1^ at 35 °C, indicating endothermic adsorption but with lower overall capacity than LTH. Langmuir shows poorer fit (*R*^2^ = 0.97–0.98) with uncharacteristically high *q*_0_ values (56.53–69.15 mg g^−1^) and large errors (±10.08), reflecting model misapplication to a heterogeneous surface. Freundlich *n* values of 1.41–1.53 (>1) indicate favorable adsorption on heterogeneous sites, with *K*_F_ increasing from 2.30 to 3.88 with temperature. Redlich–Peterson *g* values of 3.13 and 2.085 deviate substantially from unity, confirming non-Langmuir behavior; the extremely small “*a*” parameters (10^−6^–10^−8^) indicate weak lateral interactions. Hill model *n* values (1.507–1.467) match Sips heterogeneity factors, confirming positive cooperativity—adsorption at one site facilitates binding at neighboring sites. Temkin B values (10.68–11.58) with low K_T (0.346–0.570) suggest different interaction energies than LTH.

The contrasting isotherm behaviors directly reflect structural differences. LTH, with its uniform layered architecture and well-defined interparticle exchange sites, provides energetically equivalent binding positions—hence Langmuir behavior. LTO, comprising intimately mixed ZnO, CuO, and ZnFe_2_O_4_ phases, presents diverse surface sites with varying affinities—hence Sips heterogeneity. The cooperative adsorption (*n* > 1) in LTO suggests that dye molecules bound to one oxide phase may facilitate binding on adjacent phases through surface interactions or structural reorganization during memory effect reconstruction. The lower capacities of LTO reflect its reduced surface area, while the persistent heterogeneity confirms that phase mixing creates a fundamentally different adsorption landscape than the pristine layered precursor.

##### 3.2.5.4 Thermodynamic studies

The thermodynamic parameters for Indigo Carmine adsorption onto LTH and LTO were evaluated over the temperature range of 288.15–308.15 K, with values summarized in Table 3 (Fig. 5f). For LTH, the negative Δ*G*° values (−1.25 to −4.15 kJ mol^−1^) confirm spontaneous adsorption, with the increasingly negative trend at higher temperatures indicating that the process becomes more favorable with elevated temperature (Fig. 5f). The positive enthalpy change (Δ*H*° = +40.42 kJ mol^−1^) indicates endothermic physisorption and suggests that uptake is governed by physical interactions, primarily electrostatic attraction to the positively charged external surfaces and edge sites of the layered structure. The positive entropy change (Δ*S*° = +144.5 J mol^−1^ K^−1^) reflects increased disorder at the solid–liquid interface during adsorption. This entropy gain arises from the displacement of surface-bound water molecules and carbonate anions from accessible edge sites as IC molecules bind to external surfaces, rather than from bulk interlayer intercalation—consistent with the large molecular size of IC relative to the interlayer spacing.

**Table 3:** Thermodynamic parameters

Adsorbent	Temperature (∘C)	*T* (K)	ln *K*_c_	Δ*G*∘ (kJ mol^−1^)	Δ*H*∘ (kJ mol^−1^)	Δ*S* (J mol^−1^ K^−1^)
LTH	15	288	0.488	−1.25	+40.42	+144.5
	20	293	0.775	−1.84		
	25	298	1.152	−2.7		
	30	303	1.324	−3.20		
	35	308	1.586	−4.15		
LTO	15	288	−1.045	+2.57	+23.5	+72.75
	20	293	−0.89	+2.129		
	25	298	−0.8	+1.84		
	30	303	−0.575	61.39		
	35	308	−0.406	+1.11		

For LTO, Δ*G*° values are positive at all studied temperatures (+2.57 to +1.11 kJ mol^−1^) but decrease progressively with increasing temperature. This apparent non-spontaneity reflects the material's limited adsorption capacity (*q*_m_ = 35.56 mg g^−1^ at 25 °C), which results in a small distribution coefficient (*K*_c_ = *q*_e_/*C*_e_) under the experimental conditions. The adsorption process remains feasible because the initial concentration gradient drives uptake until surface saturation, consistent with the observed 40% removal at equilibrium. The critical thermodynamic information lies in the temperature dependence: Δ*G*° becomes less positive as temperature rises, confirming endothermic adsorption. The positive Δ*H*° (+23.5 kJ mol^−1^) falls within the physisorption range, while the positive Δ*S*° (+72.75 J mol^−1^ K^−1^) indicates entropy gain from release of surface-bound water molecules and structural reorganization associated with memory effect reconstruction on the heterogeneous oxide surface comprising ZnO, CuO, and ZnFe_2_O_4_ phases. The lower Δ*H*° magnitude compared to LTH reflects weaker adsorbate–adsorbent interactions, consistent with reduced surface area. The excellent linear correlation (*R*^2^ > 0.99) for both materials confirms reliable parameter estimation.

The contrasting thermodynamic parameters thus reflect mechanistic differences: LTH exhibits energetically favorable electrostatic attraction to external surfaces and edge sites of its high-surface-area layered structure, while LTO undergoes entropy-driven uptake on a heterogeneous oxide surface with limited capacity, where adsorption proceeds despite positive Δ*G*° values due to concentration driving forces.

#### 3.2.6 Adsorption in binary systems

**Fig. 6:**
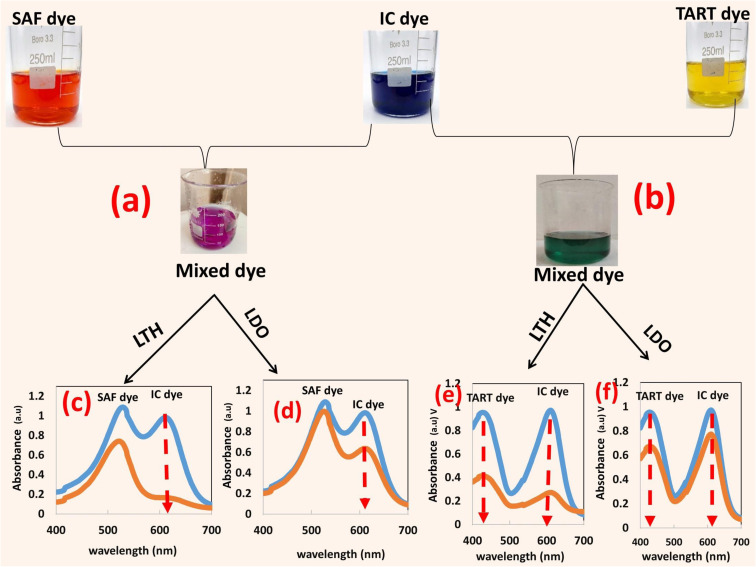
Adsorptive removal of IC dye in binary dye systems (a) indigocarmine and safranin, (b) indigocarmine and tartrazine, (c and d) IC and SAF at two concentration levels, (e and f) IC and TART at two concetration levels.

The selectivity and competitive behavior of both materials were evaluated in binary mixtures containing Indigo Carmine (anionic) with safranin O (cationic) and IC with Tartrazine (anionic), with results presented in Fig. 6a–f that clearly show how IC preferentially adsorbs relative to the co-existing dyes.

In the IC + safranin O system, both materials preferentially adsorb IC, but the extent and mechanism differ. LTH exhibits strong IC uptake with negligible safranin O removal, the slight decrease in safranin O absorbance after contact arises from removal of IC's spectral tail overlapping the safranin region, not genuine cation adsorption. The positively charged LTH surface (+16 mV at pH 6) electrostatically attracts anionic IC while repelling cationic safranin, creating effective charge-based separation. LTO shows reduced IC uptake compared to LTH and marginally more safranin O signal decrease, but the latter remains minor and largely attributable to the same spectral artifact. LTO's lower surface charge (+7 mV at pH 6) weakens electrostatic discrimination, allowing possible minor cation interaction with negatively charged oxide domains or through π–π interactions, but anionic IC remains the primary target adsorbed *via* memory effect reconstruction and surface complexation on ZnO, CuO, and ZnFe_2_O_4_ phases.

In the IC + tartrazine (anionic–anionic) system, competition for available sites may occur with both materials. LTH attracts both anionic dyes electrostatically, leading to likely competition for external surface and edge sites. The near complete IC removal reveals that IC outperforms tartrazine due to the high affinity (high Langmuir constant) and strong interaction between its sulfonate groups and the LTH surface, as reflected in greater IC peak reduction. LTO shows similar competition but with different mechanism, both IC and tartrazine dyes compete for surface complexation sites on oxide phases and for incorporation during memory effect reconstruction. The limited total sites force competitive occupation, with IC retaining slight preference due to its higher affinity for oxide surfaces. Both materials show slightly reduced uptake for each dye compared to single-dye systems, confirming competitive inhibition where available binding positions are shared between the two anionic species.

#### 3.2.7 Proposed dye removal mechanism

The adsorption mechanisms of Indigo Carmine onto LTH and LTO were elucidated by integrating spectroscopic, microscopic, and modeling evidence.

**Fig. 7:**
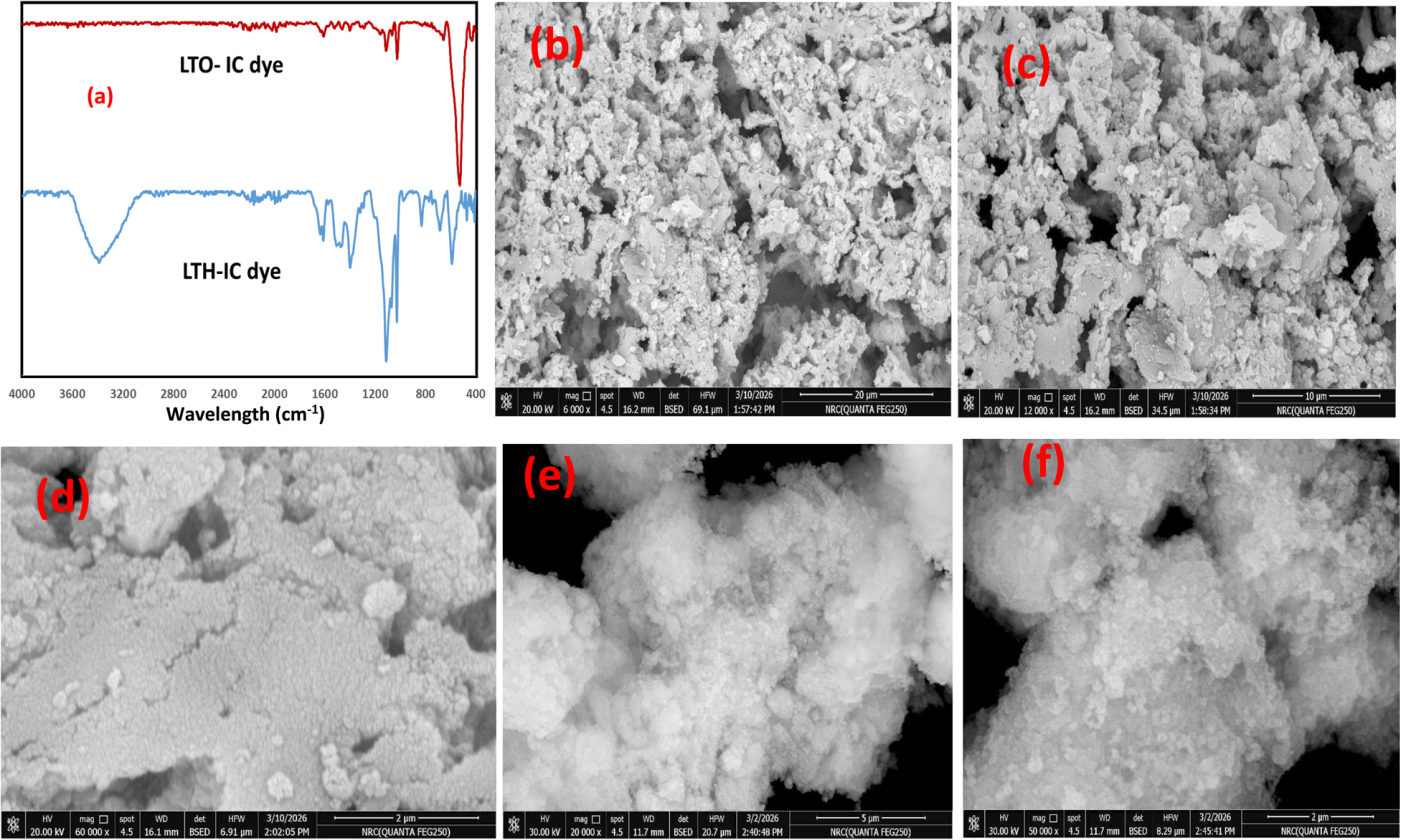
(a) FTIR of LTH and LTO after adsorption of IC, (b–d) FESEM of LTH after adoption of IC, and (e and f) FESEM of LTO after adoption of IC.

For LTH, FTIR after adsorption reveals new bands at 1651 cm^−1^ and 1232 cm^−1^—unambiguous signatures of IC's C=O and sulfonate groups (Fig. 7a). More critically, carbonate bands at 1457 and 1389 cm^−1^ diminish but do not disappear. This partial reduction signals that carbonate displacement occurs only at crystallite edges where IC can access,^44^ not throughout bulk interlayers—consistent with IC's molecular dimensions (∼1.5 nm) exceeding the 0.75 nm gallery height measured by XRD. The O–H stretching region broadens and shifts from 3426 to 3429 cm^−1^, implicating surface hydroxyls in hydrogen bonding. Modifications below 1000 cm^−1^, particularly in M–O vibrations at 1069, 1027, and 1009 cm^−1^, indicate surface complexation at edge metal sites.^46^ FESEM visually corroborates this surface-localized mechanism: hexagonal plates retain intact morphology with no swelling or exfoliation, yet smooth surfaces become roughened and edges blurred by a visible adsorbed coating—structural preservation despite clear dye coverage (Fig. 7b–d).^44^ Kinetics confirm rapid initial uptake (PFO, *R*^2^ = 0.99) consistent with accessible external sites, while Langmuir isotherm fit (*R*^2^ = 0.99, Sips *n* ≈ 1) confirms energetically equivalent binding positions on homogeneous surfaces. Zeta potential (+16 mV at pH 6) drives electrostatic attraction, and the large entropy gain (Δ*S*° = +145 J mol^−1^ K^−1^) reflects displacement of surface-bound species from accessible edges, not bulk interlayer release.

For LTO, FTIR after adsorption, similarly displays IC-characteristic bands around 1650 cm^−1^ and 1200–1250 cm^−1^, confirming dye uptake (Fig. 7a). Crucially, the low wavenumber region where M–O vibrations of ZnO, CuO, and ZnFe_2_O_4_ phases appear shows significant perturbation, direct evidence of coordination between residual IC sulfonate groups and coordinatively unsaturated metal centers on oxide surfaces. The O–H stretching region shows less recovery than expected, consistent with partial rehydroxylation during memory effect reconstruction. FESEM reveals dramatic morphological transformation: the initially open, highly porous network of nanoparticles with sharp, frizzy edges becomes compact and visibly coated (Fig. 7e and f). Deep structural voids present before adsorption appear substantially filled; interparticle porosity diminishes noticeably. At higher magnification, a dense fuzzy blanket covers the oxide aggregates, and flattened plate-like features emerge from the granular mass—direct visual evidence of memory effect activation, where LTO rehydrates and partially reconstructs layered domains, incorporating IC into reforming galleries. Kinetics show faster initial rate (*k*_1_ = 0.162 min^−1^) reflecting rapid access to macroporous network, but lower capacity due to limited sites. Sips isotherm fit with heterogeneity factor *n* > 1 confirms diverse binding sites on three oxide phases with positive cooperativity. Positive Δ*G*° values decreasing with temperature reflect concentration-driven uptake on low-capacity heterogeneous surface, while positive Δ*S*° (+72.9 J mol^−1^ K^−1^) arises from surface hydration changes and structural reorganization during reconstruction.

**Fig. 8:**
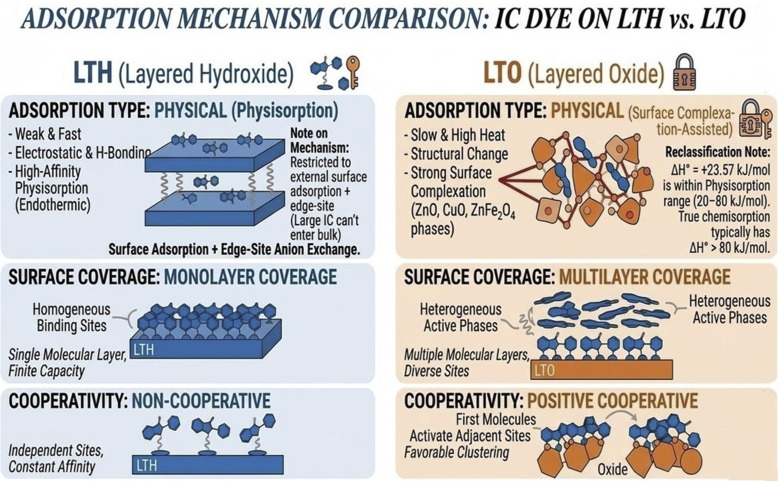
Adsorption mechanism comparison on LTH and LTO.

The integrated evidence confirms two distinct pathways. LTH binds IC through electrostatic attraction to positively charged surfaces, hydrogen bonding with hydroxyls, and edge-localized carbonate displacement—all confined to external surfaces and crystallite margins. LTO operates synergistically: initial pore-filling within its macroporous network rapidly sequesters IC, followed by surface complexation on ZnO, CuO, and ZnFe_2_O_4_ metal centers, accompanied by partial layered reconstruction *via* memory effect that entraps additional dye within reforming interlayer regions. Both mechanisms reflect the high affinity of this ternary system for anionic pollutants. This removal mechanism is schematically represented in Fig. 8 for both LTH and LTO.

#### 3.2.8 Comparative performance and mechanistic analysis

When comparing the adsorption performance of Zn–Cu–Fe LTH and LTO with other reported adsorbents for Indigo Carmine, several meaningful differences emerge that clarify the contribution of the present work (Table 4). Most published materials—including Bi_2_O_3_-doped MgO,^51^ MgFe_2_O_4_,^52^ Moringa oleifera nanosorbent,^50^ and graphene nanodots^57^—achieve IC removal through general mechanisms such as electrostatic attraction, surface complexation, or π–π interactions, yet they rarely provide direct structural evidence to confirm the proposed pathway. In contrast, the present LTH demonstrates that despite its well-ordered layered architecture, the bulky IC molecule is entirely excluded from the interlayer galleries, as confirmed by unchanged XRD basal spacing and intact plate morphology after adsorption, forcing uptake to occur exclusively on external surfaces and edge sites. This finding differs from the common assumption in the literature that layered materials remove anionic dyes *via* interlayer exchange. Regarding kinetics, montmorillonite clay^55^ shares with the present LTH a preference for pseudo-first-order behavior, whereas most other materials—including MgFe_2_O_4_ (ref. 52) and AlCoFe-LDH/C^56^—report PSO kinetics, suggesting that physisorption-dominated mechanisms may be more relevant for IC than often recognized. Graphene nanodots^57^ report spontaneous adsorption (negative Δ*G*°) consistent with the present LTH, though the LTH provides additional mechanistic clarity regarding surface *vs.* interlayer binding. In terms of practical functionality, Bi_2_O_3_-doped MgO^51^ offers higher absolute capacity (126 mg g^−1^), and Moringa oleifera^50^ achieves faster equilibrium (30 min), but neither provides magnetic separability—an advantage uniquely offered by the present LTO, which sacrifices some capacity (45 mg g^−1^) relative to the LTH (71 mg g^−1^) to gain facile recovery. Critically, most compared studies examine only single-dye systems under idealized conditions, while the present work evaluates binary dye mixtures (IC + safranin O, IC + tartrazine) as well as interference from NaCl and humic acid—factors present in real wastewaters but absent from most previous reports.^50–58^ The dual-material system presented here (LTH for higher capacity, LTO for magnetic separation) offers operational flexibility that single-material reports typically do not provide, allowing application-specific selection rather than claiming universal superiority.

**Table 4:** Performance comparison of LTH and LTO with reported adsorbent for IC

Catalyst	Preparation methods	Weight	Mechanism	*q*_max_	Key findings	Ref.
• Zn–Cu–Fe LTH	• Coprecipitation	0.04	• Electrostatic attraction (ES) +hydrogen bonding (HB)+ edge carbonate exchange	71	• IC molecule too large to enter interlayer gallery	Current Work
					• Adsorption occurs only on external surfaces and crystallite edger	
					• Rapid initial uptake due to high positive surface charge	
					• Monolayer coverage on homogeneous surface (Langmuir-type)	
					• Pseudo-first-order kinetics (physisorption-dominated)	
					• Endothermic process	
					• Surface remains positively charged at near-neutral pH	
• Zn–Cu–Fe LTO	• Calcination at 500 °C	0.04	•Memory effect + surface complexation	45	• Complete transformation of layered structure into heterogeneous oxide composite	
					• Acquires magnetic properties (easy separation)	
					• Lower surface area but larger pores	
					• Faster initial kinetics than LTH (no diffusion limitation)	
					• Heterogeneous surface with cooperative adsorption (Sips model	
					• Pseudo-first-order kinetics despite lower capacity	
					• Endothermic, entropy-driven process	
Moringa oleifera nanosorbent (nMOS)	- Oil extracted from seeds	0.8	π–π interactions, ES, HB	60	•Superior performance: 4.3× more effective than bulk form	50
	- Oil-free press cake dried at 60 °C				• High capacity: outperforms many conventional adsorbents	
	- Sieved (<51 µm)				• Fast kinetics: equilibrium reached in just 30 minutes	
	- Ball milled to <100 nm				• pH-sensitive: Best at pH 4 (below pH_pZC_ = 5)	
Bi_2_O_3_ doped MgO (MgOBi_2_) (5% Bi_2_O_3_)	—	0.02	ES + surface complexation	126	• The 5% Bi_2_O_3_ doping level gave the highest adsorption capacity among all ratios tested	51
					• The Freundlich model confirmed multilayer adsorption on a heterogeneous surface	
					• The adsorbent remained effective for 3 cycles without structural degradation	
MgFe_2_O_4_	Sol–gel method	0.1	ES + Surface complexation	46	• The isoelectric point was pH 9.5, so the surface was positively charged below this pH	52
					• Anionic IC dye was effectively adsorbed in the pH range of 4.0–9.0 due to electrostatic attraction	
					• CTAB template played a critical role by enhancing mesoporosity and preventing nanoparticle agglomeration	
Silk yarn (natural protein fiber)	Silk yarn treated with 0.5 M HCl	0.5	ES + HB	18	• The optimal adsorption conditions were pH 4.0, MLR 1 : 100, and temperature 30 °C	53
					•Adsorption was very rapid in the first 15 minutes and reached equilibrium by 60 minutes	
					• The dye bound *via* strong ionic (salt bridge) interactions between sulfonate and ammonium groups	
Zeolite from Fly Ash (ZM) (hydroxy-sodalite)	Hydrothermal activation	1.0	Electrostatic attraction + surface adsorption + intraparticle diffusion	1.2	• Zeolite synthesis successfully converted fly ash into hydroxy-sodalite	54
					• Dye removal decreased from 95% to 90% as initial concentration increased	
					• Toxicological studies with treated water showed no toxicity when ZM was used	
Montmorillonite clay	- Supplied directly from Bafra, Turkey	0.05	ES + interlayer interaction	40	• The unique finding is that pseudo-first-order kinetics gave the best fit (*R*^2^ = 0.924), while pseudo-second-order was very poor (*R*^2^ = 0.575)	55
	- Used as received (no chemical modification)				• The multi-vari chart showed that 80% removal could be achieved at high dosage and high initial concentration	
					• Montmorillonite swells in water, allowing dye molecules to enter interlayer spaces	
AlCuFe-LDH/C nanocomposite	Co-precipitation method	0.0286	ES + HB	26	• The specific surface area was 65.15 m^2^ g^−1^ with a mesoporous structure (pore size 4.49 nm)	56
					• The nanocomposite had an average particle size below 100 nm (confirmed by FE-SEM)	
					• The nanocomposite successfully removed both TZ and IC simultaneously from binary dye solutions	
Graphene nanodots (GNDs)	Modified Hummers' method to synthesize GO	0.1	π–π interactions	39	• GNDs are non-toxic, highly stable, and have excellent electrical and thermal conductivity	57
	- GO dispersed in ethanol + hydrothermal treatment				• The thermodynamic analysis confirmed the process was spontaneous (negative Δ*G*°)	

## 4 Conclusion

This comparative investigation demonstrates that calcination fundamentally redirects the adsorption pathway of Zn–Cu–Fe LTH toward Indigo Carmine removal. The pristine LTH material, despite its ordered architecture and high surface area, cannot accommodate the bulky IC molecule within its ∼0.75 nm interlayer galleries; adsorption that occurs mainly on positively charged external surfaces and partially on crystallite edges where carbonate-edge displacement may occur. Thermal transformation at 500 °C converts this layered host into a heterogeneous oxide composite composed of ZnO, CuO, and ZnFe_2_O_4_ phases, LTO, where uptake proceeds through a combination of pore-filling, surface complexation on coordinatively unsaturated metal centers, and partial reconstruction driven by the LTH memory effect. Although the calcined oxide exhibits lower overall capacity, it displays faster initial adsorption kinetics and magnetic recoverability, providing a practical advantage for post-treatment separation. Collectively, these findings indicate that for bulky IC anionic dye, LTH operate predominantly through surface-mediated adsorption rather than interlayer intercalation, while their calcined LTO derivative exploit reconstruction chemistry and heterogeneous oxide interactions to achieve uptake through an alternative pathway. This transformation expands the mechanistic framework for anionic dye remediation and highlights how controlled thermal conversion of LTH precursors can tailor both adsorption behavior and operational functionality.

## Author contributions

Mohamed Farag: formal analysis, investigation, methodology, validation, writing – review & editing. Sami A. Al-Hussain: formal analysis, investigation, writing – review & editing. Ashraf A. Mohamed: conceptualization, data curation, formal analysis, investigation, methodology, validation, visualization, writing – review & editing. Arafat Toghan: conceptualization, formal analysis, investigation, methodology, validation, writing – review & editing. Hoda A. Ahmed: formal analysis, investigation,writing – review & editing. Mohamed A. Ahmed: formal analysis, investigation, methodology, writing – review & editing. Emad M. Masoud: formal analysis, investigation, writing – review & editing. Mahmoud Adel: writing – review & editing, writing – original draft, data curation, conceptualization, formal analysis, investigation, visualization.

## Conflicts of interest

The authors declare that they have no known competing financial interests or personal relationships that could have appeared to influence the work reported in this paper.

## Supplementary Material

RA-016-D6RA03446B-s001

## Data Availability

Data for this article are available in the supplementary information (SI). Supplementary information: materials, reagents, instruments and batch adsorption and kinetic experiments. See DOI: https://doi.org/10.1039/d6ra03446b.
