# Supplementary material for: Adsorption of Indigo Carmine onto Zn–Cu–Fe layered triple hydroxides and oxides: a comparative investigation of removal mechanisms and matrix effects
Source: RSC Adv. 2026 Jun 3;16(33):30124–44. doi: 10.1039/d6ra03446b (PMC13234832; doi:10.1039/d6ra03446b)
Supplement: RA-016-D6RA03446B-s001 [file RA-016-D6RA03446B-s001.pdf]

Supplementary Information (SI) for RSC Advances.
This journal is © The Royal Society of Chemistry 2026

Adsorption of indigo carmine onto Zn-Cu-Fe layered triple hydroxides and oxides: A comparative investigation of removal mechanisms and matrix effects

Mohamed Farag[1, 3], Sami A. Al-Hussain[2], Arafat Toghan[2], Emad M. Masoud[4*], Ashraf A. mohamed[1], Mohamed A. Ahmed[1], Hoda A. Ahmed[5], Mahmoud A. Ahmed[1*]

[1] Chemistry department, Faculty of Science, Ain Shams University, Cairo-11566, Egypt

[2] Chemistry Department, College of Science, Imam Mohammad Ibn Saud Islamic University (IMSIU), Riyadh 11623, Saudi Arabia
[3] The Higher Institute for Optics Technology, Culture and Science CITY, Cairo, 17361, Egypt
[4] Department of Chemistry, College of Science in Yanbu, Taibah University, Yanbu Governorate, Saudi Arabia

*Corresponding author:

Mahmoud. A. Ahmed (mahmoud.adel.ext@veolia.com)

Materials and Reagents

All chemicals were of analytical grade and used without further purification. Zinc acetate dihydrate ($Zn(CH_3COO)_2·2H_2O$, ≥99.5%), copper(II) sulfate pentahydrate ($CuSO_4·5H_2O$, ≥99.0%), and iron(III) acetate ($Fe(CH_3COO)_3$, 97%) were used as metal precursors for the synthesis of the layered triple hydroxide (LTH). Sodium hydroxide (NaOH, ≥98%) and sodium carbonate (Na_2CO_3, ≥99.5%) served as the precipitating and pH-regulating agents while providing carbonate interlayer anions for the LTH structure. The adsorption performance of the synthesized materials was evaluated using Indigo Carmine (IC, Acid Blue 74, C.I. 73015) as the model anionic dye. For matrix interference studies, sodium chloride (NaCl, ≥99.5%) was employed to simulate high-ionic-strength conditions, and humic acid (HA, technical grade) was used to represent natural organic matter commonly present in real water bodies. All solutions were prepared with deionized water (18.2 MΩ·cm, Millipore).

Characterization of Synthesized Materials

The structural and physicochemical properties of the synthesized LTH and LTO materials were characterized using various analytical techniques. Fourier-transform infrared (FTIR) spectra were acquired using an ATI Mattson FTIR spectrometer employing the standard KBr disk method, with spectra collected over the wavenumber range of 4000–400 cm^{-1} to identify functional groups and chemical bonds present in the materials before and after adsorption. Surface morphology and elemental composition were examined using field emission scanning electron microscopy coupled with energy-dispersive X-ray spectroscopy (FESEM-EDS). High-resolution transmission electron microscopy

(HRTEM) images were obtained using a JEOL-2100 HRTEM instrument to investigate the internal structure, lattice fringes, and crystalline nature of the prepared adsorbents. Textural properties including specific surface area, pore volume, and pore size distribution were determined by nitrogen adsorption-desorption measurements at 77 K using a surface area analyzer. The specific surface area was calculated using the Brunauer-Emmett-Teller (BET) method, while pore size distribution was derived from the desorption branch of the isotherm using the Barrett-Joyner-Halenda (BJH) model. Surface charge characteristics were evaluated by zeta potential measurements over a pH range of 2.0–10.0 to determine the point of zero charge (pHpzc) of both materials. X-ray photoelectron spectroscopy (XPS) analysis was performed using a K-ALPHA XPS instrument (Thermo Fisher Scientific, USA) with monochromatic Al Kα radiation to elucidate the elemental composition and chemical states of surface species, providing insights into the adsorption mechanism and any changes occurring after dye uptake.

Adsorption Experiments

Batch adsorption experiments were conducted to evaluate the uptake behavior of Indigo Carmine (IC) onto the synthesized LTH and LTO adsorbents. A stock solution of IC with concentration 1.0×10^{-3} mol/L (that is 466 mg/L) was prepared by dissolving the required amount of dye in deionized water, and all working solutions of desired concentrations were obtained by appropriate dilution of this stock. Adsorption isotherms were investigated by varying the initial dye concentration from 5.0×10^{-6} to 7.0×10^{-5} mol/L, (that is 2.33 - 32.64 mg/L). For each experiment, 0.04 g of adsorbent was added to 100 mL of IC solution of known concentration in 250 mL Erlenmeyer flasks. The mixtures were agitated on an orbital shaker at 150 rpm and maintained at constant

temperature (298, 308, or 318 K) until equilibrium was reached. After each adsorption experiment, the solid phase was separated by centrifugation, and the residual dye concentration in the supernatant was determined using a UV-Vis spectrophotometer by monitoring the absorbance at the maximum wavelength of IC (610 nm). The amount of dye adsorbed at equilibrium, qe (mg/g), and the removal percentage (%) were calculated using standard mass balance equations.

The effect of solution pH on IC adsorption was examined across a pH range of 2.0 to 10.0. The initial pH of the dye solutions was adjusted using 0.1 mol/L HCl or NaOH solutions prior to adsorbent addition, and the experiments were carried out under the conditions described above. To investigate the influence of ionic strength, adsorption studies were performed in the presence of sodium chloride (NaCl) at concentrations ranging from 0 to 1000 ppm. Similarly, the effect of natural organic matter was evaluated by conducting adsorption experiments in solutions containing humic acid at concentrations up to 20 ppm. All experiments were performed in duplicate, and the mean values with standard deviations are reported.

Competitive adsorption behavior was investigated in mixed dye systems to evaluate the selectivity of LTH and LTO toward Indigo Carmine (IC) in the presence of coexisting dyes. Two binary systems were examined: (i) IC (5×10^{-5} M) mixed with Safranin O (3×10^{-5} M), and (ii) IC (5×10^{-5} M) mixed with Tetrazine (4×10^{-5} M). These concentrations were selected to represent realistic concentration ratios while remaining within the linear range of the corresponding UV–Vis calibration curves. Residual concentrations of the individual dyes were determined by deconvoluting the overlapping absorption spectra using absorbance measurements at the characteristic wavelengths of

IC (610 nm), Safranin O (520 nm), and Tetrazine (425 nm). The concentrations were subsequently calculated by solving simultaneous equations derived from the Beer–Lambert law. The total dye concentration in the binary mixtures was 8×10^{-5} M for the IC/Safranin O system and 9×10^{-5} M for the IC/Tetrazine system.

Kinetic studies were performed by withdrawing samples at predetermined time intervals from 0 to 80 min, and the dye concentration was measured as described above. All adsorption experiments were conducted in duplicate, and the reported values represent the mean with standard deviation.

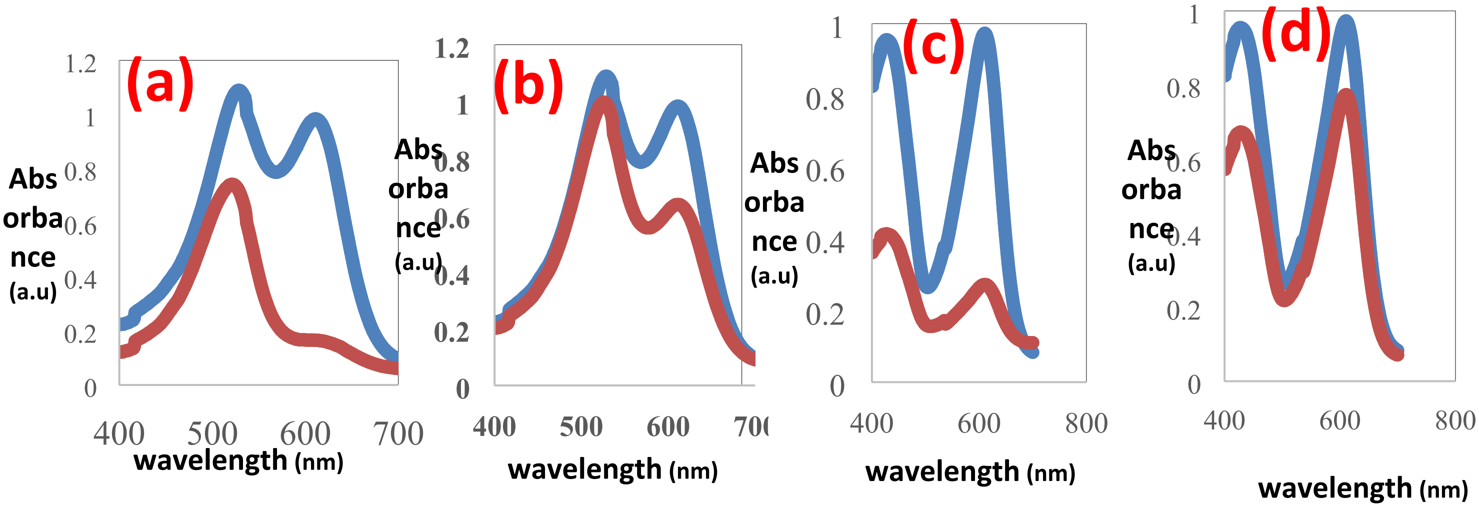

Raw UV-Vis absorbance data for binary dye adsorption experiments corresponding to Figure 7. Absorbance spectra of IC + Safranin O mixture before adsorption (blue line) and after adsorption onto LTH (red line) (a), and onto LTO (red line) (b); absorbance spectra of IC + Tetrazine mixture before adsorption (blue line) (c), and after adsorption onto LTH (red line) and LTO (orange line) (d).
